# Supplementary material for: Conditional chemoconnectomics (cCCTomics) as a strategy for efficient and conditional targeting of chemical transmission
Source: eLife. 2024 Apr 30;12:RP91927. doi: 10.7554/eLife.91927 (PMC11060718; doi:10.7554/eLife.91927)
Supplement: Figure 3—source data 3. [file elife-91927-fig3-data3.zip › Figure 3 source data3 part 2.zip!/Figure 3-Source data 3-part2/1060f2-1-CRISPResso_Report_gl5umwyq.zip!/CRISPResso_on_1060f2_3/2a.Nucleotide_percentage_quilt.pdf]

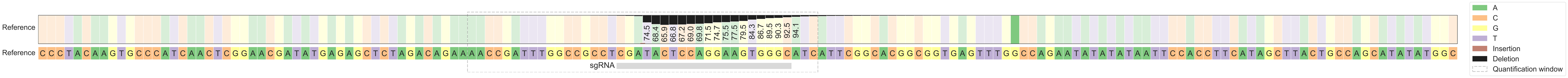
Reference
74.5
68.4
65.9
66.8
67.2
69.0
69.8
71.5
74.7
75.5
77.5
79.5
84.3
86.7
89.5
90.3
92.5
94.1
Reference
CCCTACAAGTGCCCATCAACTCGGAACGATATGAGAGCTCTAGACAGAAAACCGATTTGGCCGCCTCGATACTCCAGGAAGTGGGCATCATTCGGCACGGCGGTGAGTTTGGCCAGAATATATATAATTCCACCTTCATAGCTTACTGCCAGCATATATGGC
sgRNA
A
C
G
T
Insertion
Deletion
Quantification window
